# Strengthening Analysis of Ni-Sn Alloy Layer by Double Alloying Method Combined with First-Principles Calculation

**DOI:** 10.3390/nano12234127

**Published:** 2022-11-22

**Authors:** Lingyan Zhang, Kai Xu, Tulu Liang, Jin Shi

**Affiliations:** 1 Research Center for Intelligent Information Technology, Nantong University, Nantong 226019, China; 2 School of Information Science and Technology, Nantong University, Nantong 226019, China

**Keywords:** nanocrystalline, high-current pulse electron beam (HCPEB), mechanical alloying (MA), surface alloying, strengthening mechanism

## Abstract

Surface modification improves the performance of materials in practical applications. In this paper, mechanical alloying pretreatment is adopted. Advanced non-equilibrium processing of a high-current pulse electron beam is then used to obtain an Sn-rich Ni-Sn alloy layer, characterized by a higher surface hardness than the initial pure nickel layer. In addition, the atomic structure of the Sn-doped nickel substrate in the alloy layer is simulated, and the improvement of the alloy layer is analyzed based on the first principle. Mechanical alloying and electron beam irradiation form fine and even nanocrystals on the surface of the nickel. Sn doping is also important for enlarging the lattice and increasing the stress.

## 1. Introduction

The requirements of equipment material performance are continuously increasing with the improvement of modern industrial production technology. Improving the performance of equipment components is often costly, and component damage often starts at the surface. Therefore, this problem can be solved by improving the physical and chemical properties of the material surface [1,2,3]. Some traditional surface modification technologies, such as cladding, spraying, and penetration, cannot be significantly improved in practical application, due to the limitations of low solubility, poor interfacial binding, and low diffusivity [4,5]. For example, the spark plasma sintering method has been used to improve the mechanical properties of nickel, which is not suitable for maintaining the stability of the overall performance of devices [6]. Chen et al. strengthened nickel surfaces by laser shock peening. The round spots of laser shock overlap in some areas, making the surface treatment uneven [7].

A high-current pulse electron beam (HCPEB) technique is an advanced, effective surface modification approach [8,9]. On this basis, surface alloying was chosen as the modification method. The material is the solvent, and the alloying elements are solutes that form a high-performance alloy layer on the material’s surface [10,11].

After the electron beam irradiates the material surface, it induces fine crystal formation, slip, and dislocations. The formation of these defect structures improves the surface free energy and lattice distortion energy, providing a greater atomic diffusion driving force [12,13]. On the other hand, rapid surface cooling and solidification after electron beam irradiation can easily form supersaturated solid solutions, improve alloying diffusion ability, and surpass the modification ability of traditional technologies [14,15].

The method of pre-coating, i.e., applying a layer of alloy powder and binder mixture on the surface of the substrate, is generally adopted. Even though this method is convenient, the binding degree is not high enough. Most of the alloy powder and binder is affected by irradiated sputtering loss, reducing the alloy rate [10,16]. This paper adopts a mechanical alloying (MA) pretreatment method. Pure nickel bulk substrate is ball-ground with Sn powder to form a dense Sn powder layer on the nickel surface. Then, the surface was irradiated by HCPEB equipment. Through the MA + HCPEB composite alloying process, high-concentration Sn alloying is performed on the nickel surface to achieve high utilization efficiency and concentration diffusion of the alloying elements and this improves the properties of the alloy layer. This idea is the core starting point and innovation of this paper.

## 2. Materials and Methods

### 2.1. Experimental Procedures

Pure nickel materials are widely used in aerospace, electronic devices, military applications, and the food industry. Sn can control the vacancy, influence the precipitate interface, and manipulate the evolution of the precipitate phase. Therefore, the mechanical properties of Ni can be further improved by forming an Ni-Sn alloy. The base material used in this study is an industrial pure Ni plate cut into cubes with side lengths of 10 mm. The bulk Ni substrate samples are then annealed at 700 °C for 4 hours and mechanically ground and polished. Sn powder with 99.9% purity was selected as the alloy powder. 

The traditional presetting method involves mixing the binder and alloy powder to form a slurry. Then, the slurry is sprayed onto the surface of the substrate, but the resultant force is not strong enough. The first key method of this experiment involves mechanical alloying to fully mix the pure nickel block and Sn alloy powder by ball milling. The ball mill used is the YXQM-2L vertical planet nano ultra-fine grinding ball mill. The tank contains 3 sizes of 300 g stainless steel grinding balls, 30 g of Sn powder, and 10 pure nickel block samples. The tank is prefilled with high-purity argon gas to prevent oxidation in the ball-grinding process. The milling time is three hours, and the rotation speed is 300 r/min. 

After ball milling, the Sn powder will stick to the surface of the nickel block. A continuous, compact, and well-bonded powder composite layer with a thickness of 0.1 mm is formed on the surface of the pure nickel block, and the sample is named the Ni-Sn sample. The parameters listed in Table 1 are used to irradiate the surface of the Ni-Sn samples for 10, 20, and 30 pulses.

Microstructures of the Ni-Sn samples were characterized by an FEI Nova-Nano450 scanning electron microscope (SEM, Hillsboro, OR, USA) equipped with an energy dispersive spectrometer (EDS), at 15 kV accelerating voltage. Phase evolution was examined by a RigakuD/max-2500/pc X-ray diffractometer (XRD, Tokyo, Japan) with CuKa radiation, an NaI crystal scintillation counter detector, 285 mm diffractometer radius, 0.02° step size and 5°/min step time. Microhardness was measured by an HVS-1000 testing instrument (Beijing, China) with a load of 0.098 N (10 g) applied for 15 s, and in order to reduce random errors, six test points were installed in each sample. The maximum and minimum values were removed, and then the average of the remaining four readings was adopted as the microhardness value.

### 2.2. Theoretical Calculation Method

The internal mechanism was calculated and analyzed according to the above-described experimental procedures and combined with the first principle to optimize the alloy design.

Firstly, the atomic model was constructed using Materials Studio software (version 7.0; Accelrys Software Inc.: San Diego, CA, USA, 2013). Then, the density functional theory (DFT) calculation was performed using the Vienna Ab initio Simulation Package (VASP, version 6.1; University of Vienna, Austria, 1993) program [17]. The GGA-PBE functional was used as the exchange-correlation potential [18]. The electron wave function was expanded using a plane with a plane wave truncation energy (E_cut_) of 500 eV. The convergence value of the system’s total energy was 10^−5^ eV/atom, and the Helmann–Feynman force on each atom was no higher than 0.01 eV/Å [19]. 

## 3. Results

### 3.1. XRD Patterns

**Figure 1:**
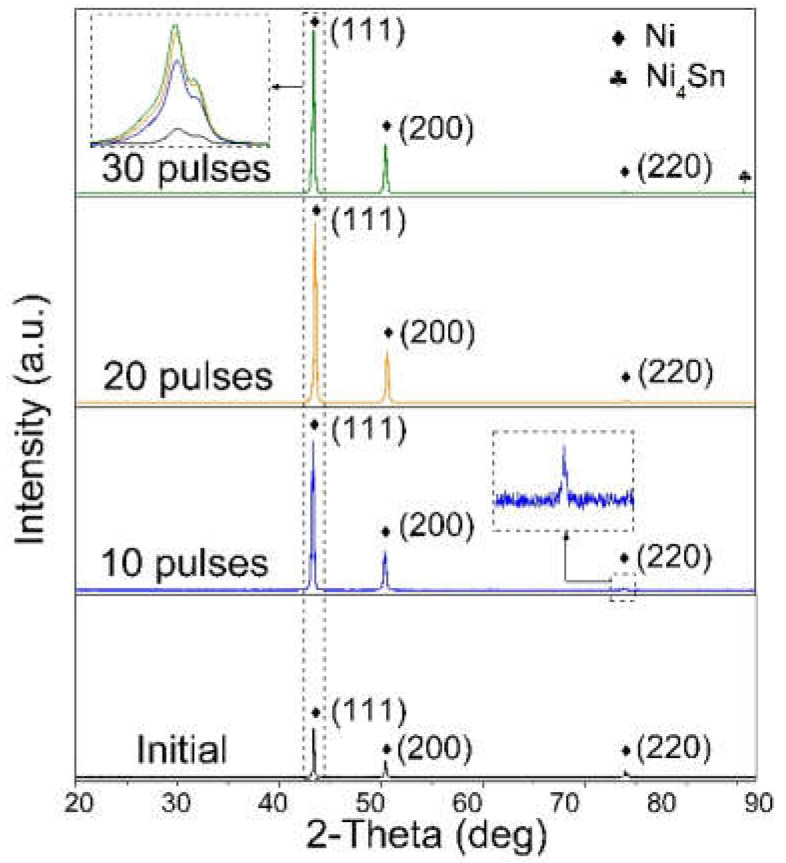
XRD patterns of Ni-Sn samples before and after HCPEB irradiation.

Figure 1 shows the XRD patterns of the Ni-Sn samples before and after HCPEB pulse irradiation. The initial sample was a pure nickel sample, and only the diffraction peak of Ni was observed. After HCPEB, the Ni peaks shifted slightly to a lower angle, indicating that Sn atoms with larger atomic radii were doped into the pure nickel substrate. In particular, the alloy compound Ni_4_Sn was detected in 30 irradiated samples. It was found that the half-peak width of nickel increases slightly with the irradiation pulses, which indicates grain refinement on the surface of the irradiated samples. Therefore, the surface of the alloyed sample was carefully observed.

### 3.2. SEM Characterization

**Figure 2:**
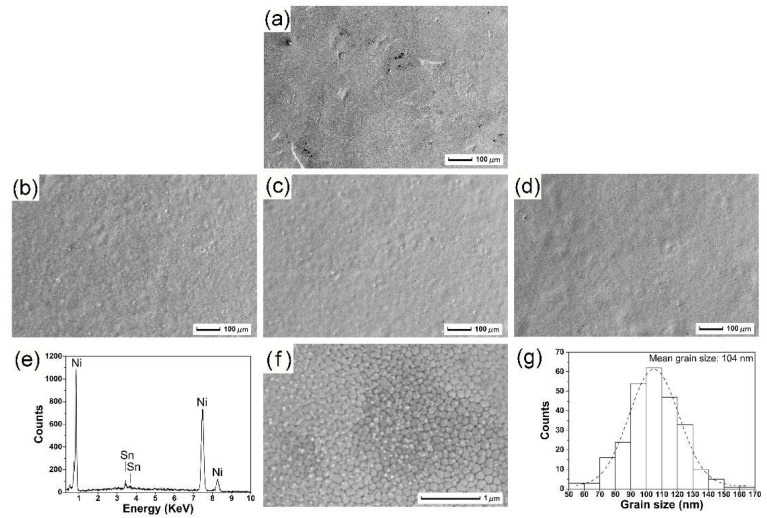
(**a**) SEM morphology of Ni surface after ball milling. Surface morphology of Ni-Sn alloyed samples after (**b**) 10 pulses, (**c**) 20 pulses, and (**d**,**f**) 30 pulses of HCPEB irradiation. (**e**) EDS analysis of Ni-Sn alloyed samples after 30 pulses. (**g**) Normal distribution curve of nanocrystal sizes.

Various craters formed on the surface of the nickel substrate after ball milling due to collisions, as shown in Figure 2a. On the basis of mechanical pre-alloying, the surface of nickel was irradiated using electron beam equipment. The surface topography of the irradiated samples was observed by a microscope. By using a scanning electron microscope, it was observed that electron beam irradiation produced many craters and particles on the sample surface. The formation of the craters can be attributed to the melting of the sample surface caused by HCPEB irradiation, as shown in Figure 2b. Moreover, with an increase in irradiation pulses, the surface of the Ni-Sn sample becomes slightly concave and convex, while the overall surface is smooth and flat, as shown in Figure 2c,d. This is because multiple irradiations lead to additional energy deposition, and the impurity eruption on the subsurface reduces the probability of crater nucleation. The surface fusion pits fuse to reduce the number of pits and achieve the effect of surface polishing and even purification.

Figure 2e shows the EDS scanning analysis results of the sample surface irradiated by 30 pulses. It can be observed that the area contains the 96.37 wt.% substrate element Ni and 3.63 wt.% alloy element Sn. This indicates that the alloy elements successfully dissolved into the substrate. Additionally, many nanocrystals were found on the surface of the sample irradiated for 30 pulses by local magnified observation, as shown in Figure 2f. 

The formation of nanocrystals can be attributed to the sample surface being rapidly heated and melted while the substrate remains cold. Hence, a large temperature gradient is formed between the pulsing surface and the substrate. Consequently, heat is rapidly transferred through the substrate via conducted, rapid surface solidification that melts the metal, while the grain fails to timely increase in size, due to the fast cooling rate (10^5^–10^7^ K/s). As a result, the grains on the irradiated surface can be significantly refined after HCPEB treatment. The normal distribution curve of nanocrystal sizes is shown in Figure 2g, with an average size of 104 nm.

**Figure 3:**
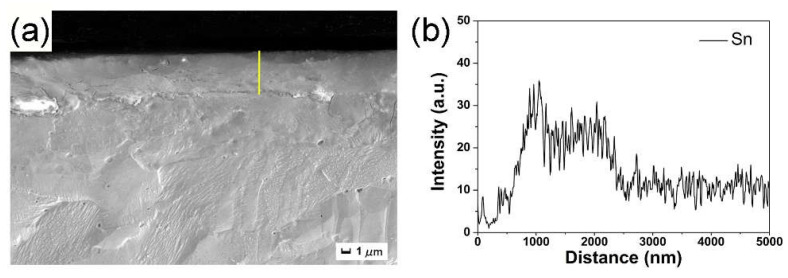
(**a**) Cross-sectional SEM images of the Sn alloyed sample irradiated by 30 pulses, and (**b**) EDS line analysis of the yellow line in (**a**).

Figure 3 shows an SEM image of the cross-section of the Ni-Sn alloyed sample irradiated by HCPEB and the EDS line scanning curve of the section. The EDS analysis results of the yellow line in Figure 3a are shown in Figure 3b. It can be observed that an Sn-rich alloy layer with a thickness of approximately 2 μm is formed on the surface of the Ni substrate after irradiation. The entire alloy layer is uniform, smooth, and compact, and metallurgical bonding with the substrate occurs.

### 3.3. Hardness Measurements

**Figure 4:**
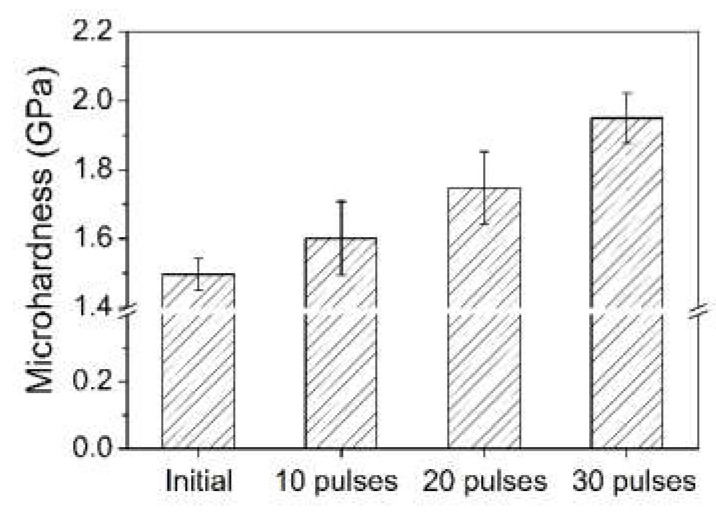
Microhardness measurements of pure nickel and Ni-Sn samples after HCPEB irradiation.

Figure 4 shows the hardness histogram of Ni-Sn alloyed samples before and after HCPEB irradiation. The surface hardness of Ni-Sn alloyed samples increases with the number of irradiation pulses. Furthermore, the surface hardness after 30 irradiation pulses reached 1.95 GPa. The surface hardness increases after repeated irradiation primarily due to many fine grains in the alloy layer, while the grain boundary structures impede dislocation movement. In addition, an Sn atom with a larger atomic radius is doped into the nickel lattice, the atomic spacing decreases, the atomic bonding ability is enhanced, and the strengthening effect is improved.

### 3.4. VASP

**Figure 5:**
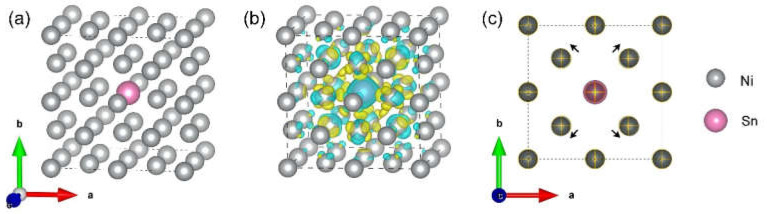
(**a**) Supercell structure of Ni_31_Sn, (**b**) differential charge density diagram of Ni_31_Sn; (**c**) atomic displacements before and after doping.

Pure nickel has a face-centered cubic structure, its first-order protocell contains four atoms, and the space group is Fm-3m. As shown in Figure 5a, a 2 × 2 × 2 pure nickel supercell structure with 32 atoms was constructed. The pink atom is the displacement site of the alloying element Sn with a displacement ratio of 1:31, simulating the possible structure of an alloying element doped into the substrate. After replacing Ni with Sn atoms, an obvious electron overlap and charge exchange with the surrounding nickel atoms can be observed, as shown in Figure 5b. The blue part of the diagram shows a decrease in charge density and the yellow part shows an increase in charge density. The doping of Sn atoms causes the electrons to rearrange themselves and gather between Ni and Sn atoms, increasing the interaction. Figure 5c shows the atomic displacements before and after doping at the (00 $\frac{2}{c}$) position. The cross in the figure marks the original nickel atom. After the middle position is taken over by Sn, the nickel atoms move outwards, which is consistent with the results of XRD.

## 4. Discussion

The surface of the mechanically pre-alloyed nickel substrate is irradiated by an electron beam. The non-equilibrium process produces various defects, and the strain field that generates stress is formed in proximity, which also hinders the dislocation movement and increases the surface hardness. Due to the increase in grain boundaries and the refinement induced by the mechanical alloy and electron beam, the formation of many defect structures promotes the diffusion of Sn atoms. Consequently, the solid solubility of Ni (Sn) solid solutions is improved. In addition, an increase in hardness can also be attributed to the lattice distortion caused by Sn atom doping. This, in turn, increased the dislocation resistance. Hence, higher external stress is required to overcome the elastic strain of dislocation interaction.

First-principles calculations can be combined with experiments to provide theoretical analysis and design predictions for experimental phenomena. Future research will focus on additional investigations regarding thermodynamic and dynamic simulations.

## Figures and Tables

**Table 1:** Parameters of HCPEB process.

Accelerated Voltage	Current Pulse Duration	Energy Density	Beam Diameter	Pulse Interval	Vacuum
27 keV	1.5 μs	4 J/cm^2^	60 mm	8 s	5 × 10^−3^ Pa

## Data Availability

The data presented in this study are available upon request from the first author.
